# Physiologically Based Pharmacokinetic Modeling to Understand the Absorption of Risperidone Orodispersible Film

**DOI:** 10.3389/fphar.2019.01692

**Published:** 2020-02-03

**Authors:** Fang Chen, Hongrui Liu, Bing Wang, Liuliu Yang, Weimin Cai, Zheng Jiao, Zhou Yang, Yusheng Chen, Yingjun Quan, Xiaoqiang Xiang, Hao Wang

**Affiliations:** ^1^ National Pharmaceutical Engineering Research Center, China State Institute of Pharmaceutical Industry, Shanghai, China; ^2^ Department of Clinical Pharmacy, School of Pharmacy, Fudan University, Shanghai, China; ^3^ Shanghai Chest Hospital, Shanghai Jiao Tong University, Shanghai, China; ^4^ Department of General Surgery, Shanghai Pudong Hospital, Fudan University Pudong Medical Center, Shanghai, China

**Keywords:** risperidone orodispersible film, oral cavity compartment absorption and transit model, advanced compartment absorption and transit model, GastroPlus™, oral residence time, oral transmucosal delivery

## Abstract

**Objective:**

The aim of the present study was to investigate the absorption routes as well as the potential application of the oral transmucosal delivery of risperidone orodispersible film (ODF) using physiologically based pharmacokinetic modeling.

**Methods:**

The pharmacokinetic study after intragastric (i.g.), supralingual, and sublingual administration of risperidone ODF was conducted in Beagle dogs. Then a mechanic absorption model which combined Oral Cavity Compartment Absorption and Transit (OCCAT) model with Advanced Compartment Absorption and Transit (ACAT) model for predicting the absorption routes of risperidone ODF *in vivo* was constructed using GastroPlus™. A sensitivity analysis was performed to investigate the impact of oral residence time on the *in vivo* absorption of risperidone ODF. Based on the fraction of intraoral absorption, the potential of the oral transmucosal delivery of risperidone were predicted.

**Results:**

There were no statistical differences in the AUC_0–t_ (P = 0.4327), AUC_0-∞_ (P = 0.3278), C_max_ (P = 0.0531), and T_max_ (P = 0.2775) values among i.g., supralingual, and sublingual administration of risperidone ODF in Beagle dogs. The predicted absorption percentage *via* oral mucosa at oral residence time of 2 min, 5 min, and 10 min was 7.0%, 11.4%, and 19.5%, respectively. No obvious difference was observed for the bioavailability of risperidone ODF within 10 min of oral residence time. The PBPK absorption model for risperidone could be simplified to include ACAT model solely.

**Conclusion:**

The main absorption route for risperidone ODF was the gastrointestine. The absorption percentage *via* oral mucosa was almost negligible due to the physicochemical properties of risperidone although ODF dissolved completely in the oral cavity of Beagle dogs within 2 min.

## Introduction

Orodispersible films (ODFs) are single or multilayer sheets of water-soluble polymer materials (Madhav et al., 2009; Hoffmann et al., 2011; Lam et al., 2014; Krampe et al., 2016; Foo et al., 2018). Due to the instant disintegration and release of the drug into the saliva once the film is put into the oral cavity, there is no need of water for the ingestion and it is more convenient than conventional tablets (Poston and Waters, 2007; Hoffmann et al., 2011; Lam et al., 2014). Owing to the characteristic of fast wetting, ODFs may adhere to the oral mucosa site and dissolve rapidly, so they cannot be spat out easily. Therefore, they are very suitable for special patients such as pediatric, geriatric, and psychiatric patients. They can effectively improve the clinical compliance (Poston and Waters, 2007; Krampe et al., 2016).

Risperidone, a benzisoxazole derivative, is a second-generation antipsychotics which has a high affinity for multiple receptors including 5-HT_2A_ serotonin, D_2_ dopamine, α_1_, α_2_ adrenergic, and histamine receptors (Huang et al., 1993; Megens et al., 1994; Meuldermans et al., 1994; Gong et al., 2015). Risperidone has been widely used for acute and chronic schizophrenia (Huang et al., 1993; Mannens et al., 1993; Gong et al., 2015; Narayan et al., 2016). It can also alleviate the symptoms of schizophrenia and improve the social and personal performance (Huang et al., 1993; de Leon et al., 2010). It is available as tablets, oral solutions, capsules, dispersible tablets, orally disintegrating tablets (ODTs), and ODFs in the dose strengths ranging from 0.5 to 4 mg (Khames, 2017). Taken orally, risperidone is completely and rapidly absorbed. The oral bioavailability of risperidone is about 70% and the pre-systemic metabolism yields the active metabolite of 9-hydroxy (9-OH) risperidone *via* cytochrome P450 2D6, 3A4, and 3A5 (Huang et al., 1993; de Leon et al., 2010; Shimizu et al., 2017).

Heemstra et al. employed a modified Ussing chamber to investigate the permeability of risperidone through porcine buccal mucosa at various concentrations (Heemstra et al., 2010). The results showed that risperidone could permeate through the buccal mucosa in the way of passive diffusion, indicating the potential application of the intraoral delivery for risperidone mucoadhesive gel. ODFs stick to the oral mucosa and dissolve in the oral cavity within minutes, and a portion of the drug may be absorbed directly into the bloodstream *via* oral mucosa, avoiding pre-systemic metabolism. Thus, it is necessary to understand the absorption routes that are crucial for the development of risperidone ODF.

Physiologically based pharmacokinetic (PBPK) modeling combines the system dependent physiological, anatomical, and biochemical properties, specific properties of compounds as well as the formulation parameters, providing an approach to predict the plasma concentration–time profiles from *in vitro* data (Upton et al., 2016; Lin and Wong, 2017; Hens et al., 2018). Therefore, it has gained high popularity to support decision making throughout the drug research and development.

**Figure 1:**
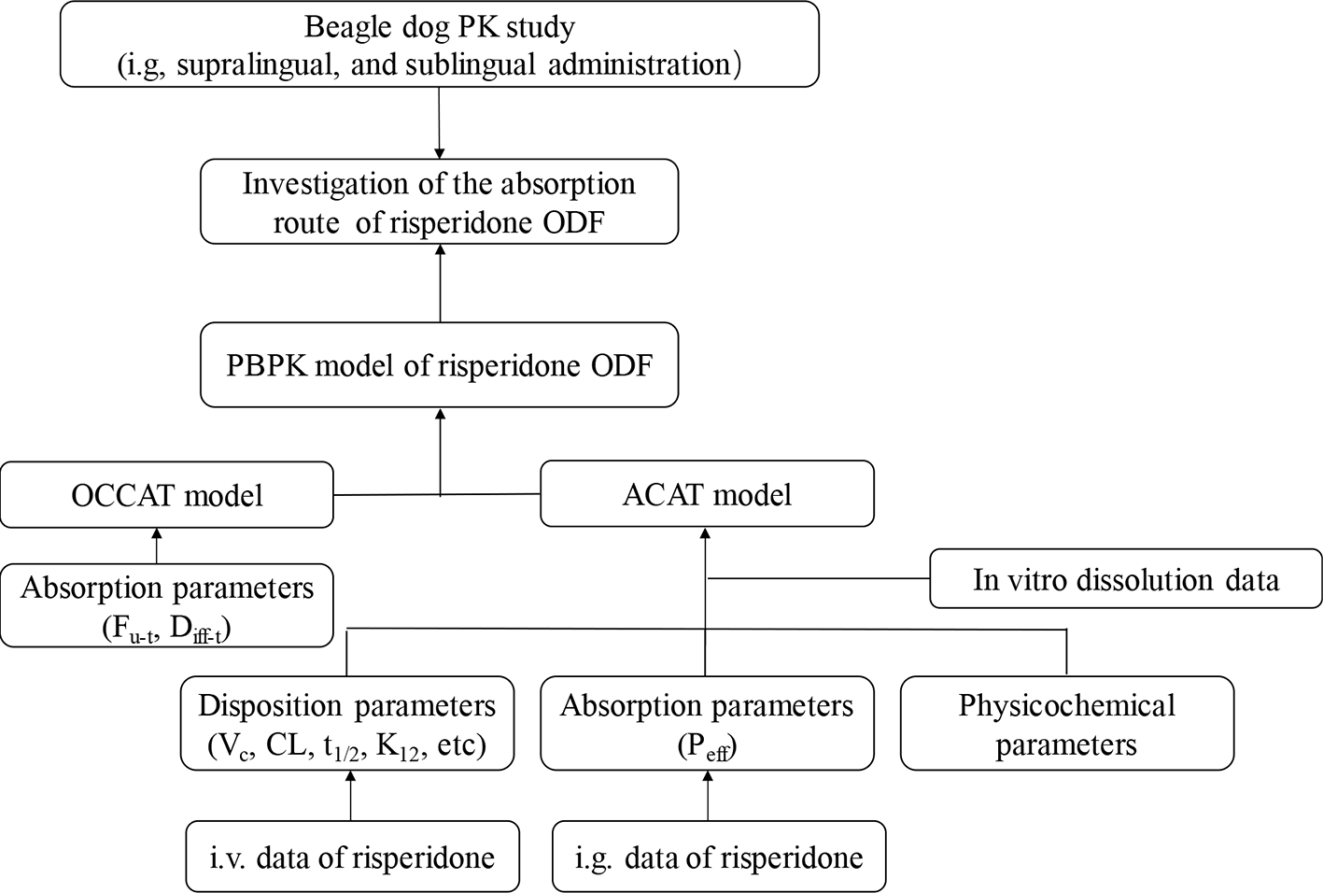
Schematic diagram of the methodology adopted.

In the present study, we aimed to investigate the application potential of the oral transmucosal delivery of risperidone ODF using PBPK model. The pharmacokinetic study was conducted in Beagle dogs to identify the difference among intragastric (i.g.), supralingual, and sublingual administration of risperidone ODF. Then a mechanic PBPK model for understanding how risperidone ODF was absorbed was constructed. To build a model, intravenous (i.v.) data were generated to obtain risperidone disposition parameter (e.g. CL and V), i.g. data were generated to understand the gastrointestinal absorption, and *in vitro* dissolution was performed to provide information to predict *in vivo* dissolution. A schematic diagram of the adopted methodology was shown in **Figure 1**. Based on the fraction of intraoral absorption, the application potential of the oral transmucosal delivery of risperidone was evaluated.

## Materials and Methods

### Preparation of Risperidone ODF

The formulation design, optimization and evaluation have been described in our previous study (Zhang et al., 2017). Briefly, risperidone ODF was prepared by solvent casting method (Foo et al., 2018).Weighted amount of risperidone was dispersed in purified water at ambient temperature under constant stirring. Subsequently, the required amount of citric acid, PEG 4000, HPMC E3, HPMC E15, aspartame, titanium dioxide, and peppermint essence were added separately under continuous grinding to obtain a homogeneous mixture. The uniform dispersion was processed in the vacuum deaeration pot to remove the air bubbles and then casted onto the backing by using a homemade coating machine with a drying temperature of 80–90 °C. The prepared films were cut into the sizes of 3 cm^2^ (2.2 cm×1.4 cm) containing 1 mg of risperidone and stored at ambient temperature for further analysis.

### *In Vitro *Dissolution Study

The *in vitro* dissolution of risperidone ODF was conducted using USP paddle at a rotation speed of 50 rpm in 500 ml of four different media, in 0.1 M HCl (pH 1.0), in acetate buffer of pH 4.0, in phosphate buffer of pH 6.8 and in water. Temperature was maintained at 37 ± 0.5 °C. Samples were collected at predetermined time intervals and filtered through a membrane filter of 0.22 μm.

The amount of risperidone was determined by a reported high-performance liquid chromatography (HPLC, Shimadzu Co. Ltd, Kyoto, Japan) method in our previous study (Zhang et al., 2017). Mobile phases consisted of acetonitrile and 5 mg/ml ammonium acetate in water (11:39, v/v) at a flow rate of 1.5 ml/min. Ten μl of the aliquot was injected into a Waters Atlantis^®^T3 C_18_ column (4.6 mm×100 mm, 3 μm, Waters Co. Ltd, Ireland) at a column oven of 40 °C. The quantity of risperidone was measured at ultraviolet wavelength of 275 nm.

### Pharmacokinetic Study

The study included a four period, 1 week wash-out, crossover, single dose by i.v., i.g., supralingual, and sublingual administration. For i.v. administration, a 0.2 mg/ml of risperidone solution was prepared by dissolving 5 mg risperidone in 25 ml of 0.05% (m/v) tartaric acid water solution. For i.g. administration, risperidone ODFs which contained 5 mg of risperidone were dissolved in 25 ml of water solution. For supralingual administration, risperidone ODFs were put onto the tongue of Beagle dogs, and for sublingual administration, risperidone ODFs were put on the bottom of the tongue. The dogs were kept still for 2 min. A dose of 1 mg/body was administrated.

Four healthy Beagle dogs (male:female, 1:1) weighting 9.04 ± 1.88 kg (Certificate No. 20150005001131), purchased from Shanghai Jambo Biological Technology Co., Ltd (Shanghai, China), were used in this study. The study protocol was reviewed and approved by the Animal Management and Ethic Committee of the China State Institute of Pharmaceutical Industry. Dogs were housed individually in stainless steel cages. Tap water was given *ad-libitum* and food was provided once daily. Dogs were fasted for 12 h before drug administration. Blank blood samples were withdrawn prior to the administration of the drugs. Subsequently, blood samples were taken from forelimb vein at 0.167, 0.333, 0.5, 0.75, 1.0, 1.5, 2.0, 3.0, 4.0, 6.0, 8.0, 12, 24, and 32 h into heparin sodium-containing centrifuge tubes which were then centrifuged for 5 min at 3,000 rpm. The supernatant was collected and stored at -20 °C in the refrigerator until further analysis.

The main pharmacokinetic parameters of maximum plasma concentration (C_max_), peak time (T_max_), area under the concentration-time curve from 0 h to the time of last measurable concentration (AUC_0-t_), and AUC from 0 to infinity (AUC_0-∞_), mean residue time (MRT), apparent volume of distribution (V_d_), elimination half-life (t_1/2_), and *in vivo* clearance (CL) were calculated by non-compartmental approach using the software of DAS 2.0 (Cheng et al., 2016). The results were summarized using their arithmetic means and standard deviations (SD). The statistically significant difference of the main pharmacokinetic parameters (C_max_, T_max_, AUC, MRT, V_d_, t_1/2_, and CL) between the administration routes were assessed by the one-way analysis of variance (ANOVA) model using the software of PASW Statistics 18 at a significance level of α = 0.05.

### Bioanalytical Method of Risperidone

A validated HPLC tandem mass spectrometry (HPLC-MS/MS) method for the determination of risperidone and the metabolite 9-OH risperidone in Beagle dog plasma were reported previously (Zhang et al., 2017). Calibration curves were constructed in the concentration range of 0.2–200 ng/ml with a lower limit of quantification (LLOQ) of 0.2 ng/ml. The intra-day precision ranged from 1.49% to 11.4% (n = 15) and the inter-day precisions ranged from 3.77% to 9.33% (n = 15). The HPLC system (Shimadzu Co. Ltd, Kyoto, Japan) equipped with a triple quadruple mass spectrometer (Shimadzu Co. Ltd, Kyoto, Japan) operating with ESI in the positive mode was used for the quantification of the analytes. Mobile phases consisted of methanol and water (35:65, v/v), both containing 0.05% formic acid and 5 mM ammonium formate. The flow rate was set at 0.3 ml/min. Ten μl of the aliquot was injected into the Inspire C_18_ column (2.1 mm×50 mm, 3 μm, Dikma Technologies Inc, Beijing, China) at a column oven of 40 °C. Multiple reaction monitoring (MRM) was utilized to determine risperidone, its metabolite 9-OH risperidone, and diphenhydramine (internal standard, IS) with transitions of *m/z* 411.10→191.10, *m/z* 427.10→207.15, and *m/z* 256.10→167.05, respectively. Direct protein precipitation using acetonitrile was used for the extraction of analytes from dog plasma.

### Model Development

The PBPK model of risperidone in Beagle dog was conducted using GastroPlus™ (version 9.7, Simulation Plus, Inc., CA, USA). Extensive and systematic literature search was performed to collect physicochemical parameters (molecular weight, solubility, pKa, and log P), blood to plasma partition coefficient (B/P) of dogs, and dog plasma unbound fraction of risperidone (f_u_) (PMDA label, 2018; Mannens et al., 1994). The diffusion coefficient of risperidone was predicted using GastroPlus™. Mean precipitation time, drug particle density, and particle size utilized default values in GastroPlus™.

The systemic clearance (CL), elimination rate constants, and volumes of distribution (V) were calculated by fitting the plasma concentration versus time profile of i.v. administration of risperidone in Beagle dogs using the empirical three-compartmental pharmacokinetic (PK) models in DAS 2.0. The obtained PK parameters were used to simulate the *in vivo* elimination of risperidone in PBPK model without further alteration.

The Advanced Compartment Absorption and Transit (ACAT) model implemented in GastroPlus™ defines the GI tract as one stomach, seven small intestine segment and one colon compartment(s), within each of which, drug can exist in several states simultaneously including unreleased, undissolved, dissolved, degraded, metabolized, and absorbed as it transits through successive compartments (Takano et al., 2006). The kinetics associated with these processes are modeled by a system of coupled linear and non-linear rate equations. The plasma concentration versus time profile of i.g. administration of risperidone in Beagle dogs was used to build the GastroPlus™ ACAT models. The effective permeability (P_eff_) value of risperidone was optimized to match the plasma concentration-time curves.

The Oral Cavity Compartment Absorption and Transit (OCCAT) model divided the oral cavity into six physiological compartments: buccal, gingival, palate, top of the tongue, bottom of the tongue, and mouth floor, which accounts for drug dissolution in saliva, diffusion through the oral mucosa, and drug absorption into the systemic circulation (Xia et al., 2015). The parameters involved in the intraoral modeling settings, such as fraction unbound in oral tissue (F_u-t_), and oral mucosa diffusivity (D_iff-t_), were estimated using GastroPlus™. The OCCAT model is linked to the ACAT model for the prediction of the percentage of absorbed drug from oral cavity.

Z-factor model (Eq. 1) in the software of GastroPlus™ was chosen to describe the *in vivo* dissolution kinetics of risperidone ODF (Takano et al., 2006). Z is a dissolution parameter, which is independent of the saturated solubility, applied amount of drug, and the volume of medium, and is determined by fitting to the *in vitro* dissolution data (Takano et al., 2006).

(1) dXd, vitrodt=z(Cs-Xd,vitro(t)Vvitro)(Xs,vitro(t)X0,vitro)23X0,vitro

where X_d,vitro(t)_ is the mass of dissolved drug at time t, r is the density of the drug, X_s,vitro(t)_ is the mass of solid drug at time t, X_0,vitro_ is the initial mass of solid drug, C_s_ is the saturated solubility of the drug, and V_vitro_ is the volume of the dissolution medium.

The predictive accuracy of the PBPK model was assessed by calculating the fold error based on the following formula:

(2) $$\text{Fold error=}\left\{\begin{array}{c} \frac{\text{observed value}}{\text{simulated value}},\text{if observed values > simulated value}\\ \frac{\text{simulated value}}{\text{observed value}},\text{if observed value > simulated value} \end{array}\right\}$$

An accurate prediction was achieved if fold error was within two (Li et al., 2009).

### Parameter Sensitivity Analysis and Regional Absorption Prediction

In OCCAT model of GastroPlus™, there are three oral transit models designed for intraoral delivery system, namely, *Normal Swallowing*, *Hold & Swallow* as well as *Hold Rinse & Swallow*. Since ODFs can maintain some time of contact in mucosal surface, the option of *Hold & Swallow* was chosen. The hold time (residence time) of the drug in oral cavity is a main factor which may affect the absorption *via* oral mucosa for rapidly-disintegrating formulations. A sensitivity analysis was performed to investigate the impact of residence time on the *in vivo* performance of risperidone ODF. Since ODFs generally release the drug immediately, we conducted the sensitivity analysis of oral residence time in a range of 0–10 min. A sensitivity factor was calculated for each systemic parameter by Eq. 3 (Hens et al., 2018).

(3) $$\text{Sensitivity factor}=\frac{\text{Maximum value}-\text{Minimum value}}{\text{Maximum value}}\;$$

The sensitivity value was between 0 and 1. The higher the value, the more sensitive it is. The percentage of absorbed drug from oral cavity was also evaluated.

## Results

### *In Vitro* and *In Vivo* Dissolution Data

**Figure 2:**
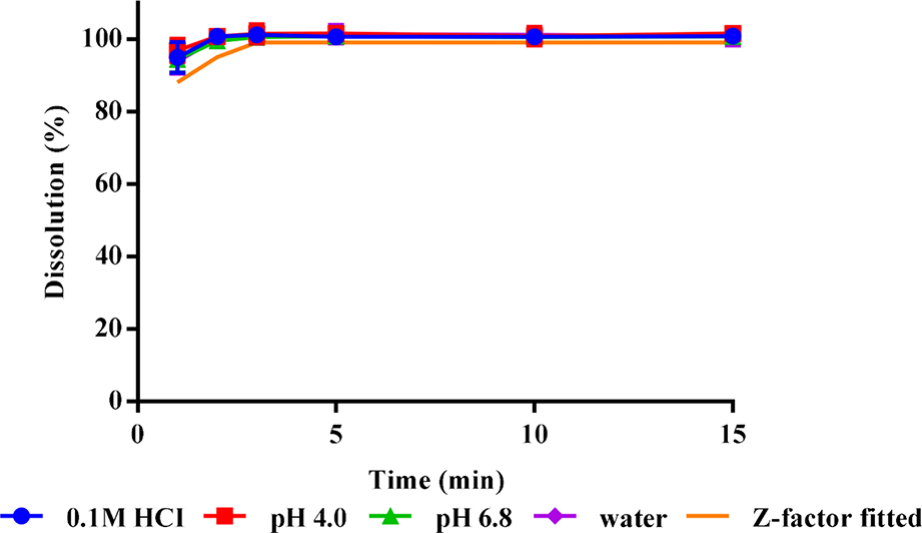
*In vitro* dissolution profiles (n = 3) and *in vivo* fitted dissolution profile for risperidone ODF.

The *in vitro* dissolution results at different test conditions and *in vivo* dissolution curve fitted by Z-factor model are shown in **Figure 2**. The ODF all achieved a complete release of its risperidone contents in different medium within 2 min. This dissolution was generally unaffected by pH at the range of 1–7.

### *In Vivo *Pharmacokinetic Data

**Figure 3:**
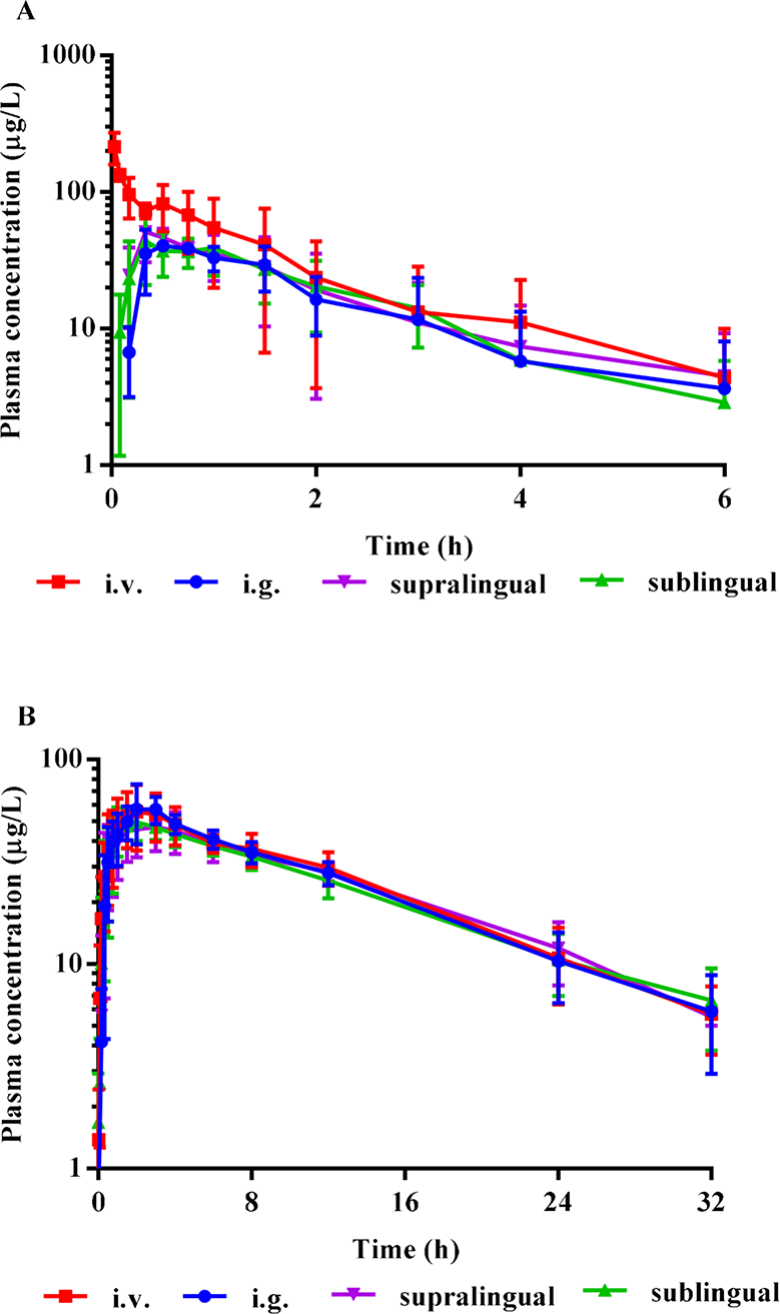
Mean plasma concentration-time curves of risperidone **(A)**, and the metabolite 9-OH risperidone **(B)** in Beagle dogs (Mean ± SD, n = 4) after i.v., i.g., supralingual, and sublingual administration of 1 mg/body. The error bars represent standard deviation (SD) from the mean.

The mean plasma concentration-time curves of i.v., i.g., supralingual, and sublingual administration of 1 mg/body risperidone in Beagle dogs are shown in **Figure 3**. The main pharmacokinetic parameters for risperidone are summarized in **Table 1**.

**Table 1:** Main pharmacokinetic parameters of risperidone (Mean ± SD) in Beagle dogs after i.v., i.g., supralingual, and sublingual administration (n = 4).

Parameters	Unit	i.v.	i.g.	Sublingual	Supralingual
AUC_0-t_	μg/L·h	177.02 ± 113.04	89.99 ± 48.73	98.89 ± 40.89	97.31 ± 54.39
AUC_0-∞_	μg/L·h	179.10 ± 115.28	91.26 ± 48.58	101.57 ± 43.34	101.87 ± 57.42
T_max_	h	0.03 ± 0	0.71 ± 0.53	0.67 ± 0.38	0.62 ± 0.58
C_max_	μg/L	214.97 ± 56.43	45.85 ± 7.60	52.57 ± 10.60	59.38 ± 7.32
MRT_0-t_	h	1.30 ± 0.80	1.71 ± 0.75	1.78 ± 0.41	1.50 ± 0.71
MRT_0-∞_	h	1.40 ± 0.89	1.79 ± 0.74	1.93 ± 0.52	1.73 ± 0.82
V_d_	L	12.77 ± 9.79	14.93 ± 1.43	17.75 ± 3.06	17.22 ± 7.70
t_1/2_	h	1.37 ± 0.82	0.92 ± 0.41	1.21 ± 0.40	1.13 ± 0.50
CL	L/h	7.69 ± 4.60	12.90 ± 5.07	11.03 ± 3.80	11.93 ± 5.24
F	%	/	57.04 ± 17.45	65.16 ± 20.16	61.98 ± 17.73

AUC_0-t_ area under the concentration-time curve from 0 h to the time of last measurable concentration, AUC_0-∞_ AUC from 0 to infinity, T_max_ peak time, C_max_ maximum plasma concentration, MRT mean residue time, V_d_ apparent volume of distribution, t_1/2_ elimination half-life, CL clearance, F relative bioavailability.

There were no statistically significant differences in the AUC_0–t_ (P = 0.4327), AUC_0-∞_ (P = 0.3278), C_max_ (P = 0.0531), T_max_ (P = 0.2775), MRT_0–t_ (P = 0.2956), MRT_0-∞_ (P = 0.5141), t_1/2_(P = 0.2719), V_d_ (P = 0.5565), and CL (P = 0.3720) values among the sublingual, supralingual, and i.g. administration routes.

### Model Development

The input parameters for GastroPlus™ modeling are summarized in **Table 2**.

**Table 2:** Summary of input parameter for GastroPlus™ Simulation of risperidone.

Parameters		Value
**Physiochemical parameters**		
Mol Weight (g/mol)		410.49
log P		3.04^a^
Compound type		dibasic base
pKa		pKa_1_ = 8.24 pKa_2_ = 3.11^b^
B/P		0.506^c^
f_u_		0.083^c^
Solubility (pH 6.8) (mg/ml)		0.9^d^
Diffusion coefficient (cm^2^/s)		0.64×10^-5e^
Mean Precipitation Time (s)		900^f^
Drug particle density (g/ml)		1.2^f^
Particle size (μm)		25^f^
**Absorption parameters**		
ACAT model	P_eff_ (10^-4^ cm/s)	5.3894^e^
OCCAT model	F_u-t_	0.15382^e^
	D_iff-t_ (cm/s)	9.383×10^-7e^
**Disposition parameters**		
First pass extraction (%)		51^e^
V_c_ (L/kg)		0.3139^e^
CL (L/h/kg)		0.5903^e^
t_1/2_ (h)		2.43^e^
K_12_ (h^-1^)		16.352^e^
K_21_ (h^-1^)		9.007^e^
K_13_ (h^-1^)		0.4625^e^
K_31_ (h^-1^)		0.4103^e^
V_2_ (L/kg)		0.56988^e^
V_3_ (L/kg)		0.35383^e^
**Dosing design**		
Dose (mg)		1
Body weight (kg)		9.0425
No. of dogs		4

log P octanol/water partition coefficient, pKa dissociation constant, B/P blood to plasma partition coefficient, f_u_ plasma unbound fraction, P_eff_ effective permeability, F_u-t_ fraction unbound in oral tissue, D_iff-t_ oral mucosa diffusivity, V volumes of distribution, V_c_ volume of distribution of the central compartment,V_2_ volume of distribution of the first peripheral compartment,V_3_ volume of distribution of the second peripheral compartment, CL *in vivo* clearance, t_1/2_ elimination half-life, K elimination rate constants.

a Taken from Drugbank.

b Taken from Pubchem.

c Taken from Ref. (Mannens et al., 1994).

d Taken from Ref. (PMDA label, 2018).

e Estimated using GastroPlus™.

f Default value of GastroPlus™

### Parameter Sensitivity Analysis

**Figure 4:**
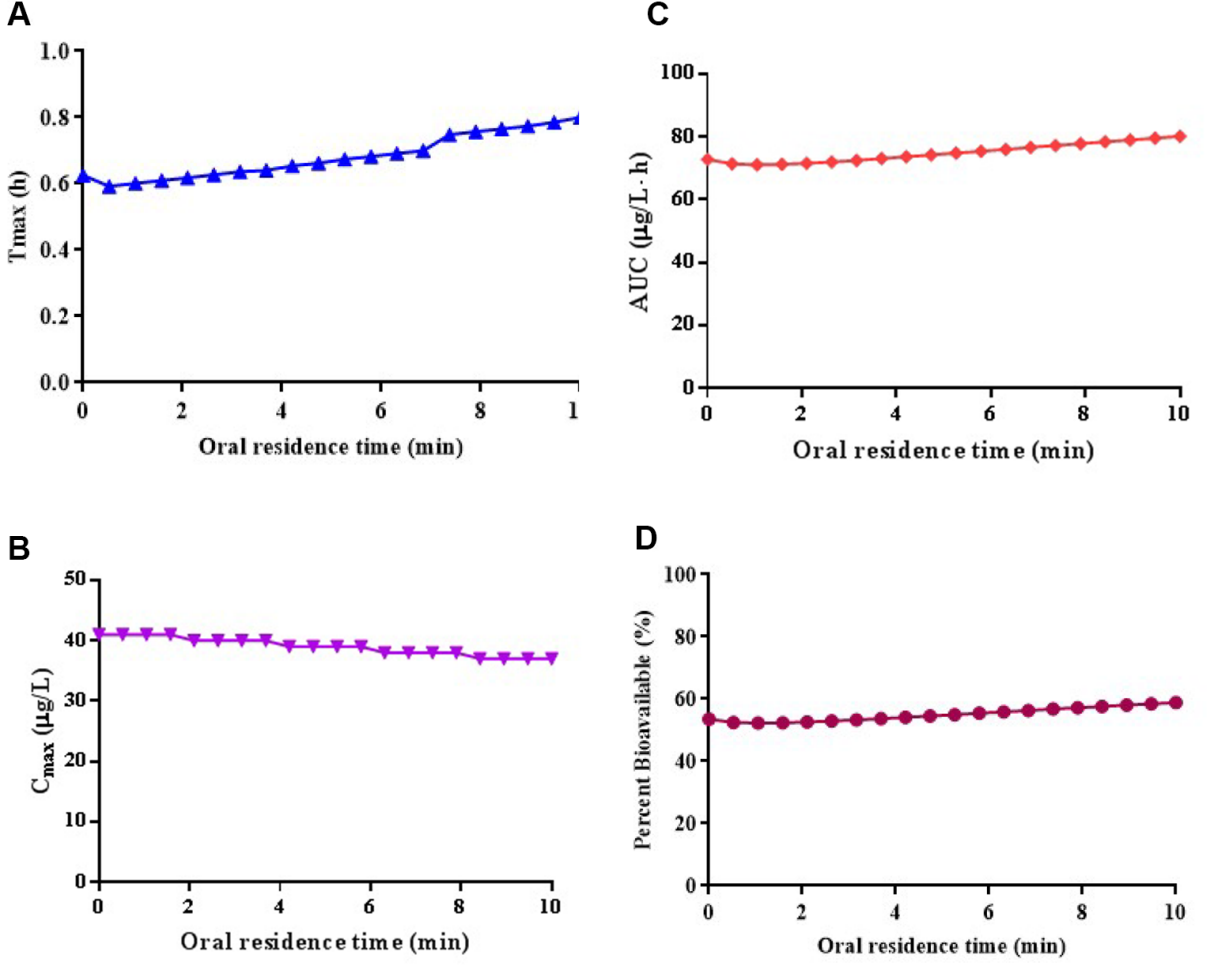
Simulated the influence of oral residence time at a range of 0–10 min on the T_max_ **(A)**, C_max_ **(B)**, AUC_0-∞_ **(C)**, and bioavailability **(D)** of risperidone ODF.

The parameter sensitivity assessed the influence of oral residence time in a range of 0–10 min on the T_max_, C_max_, AUC_0-∞_, and bioavailability of risperidone ODF, which is depicted in **Figure 4**. The C_max_, AUC_0-∞_, and bioavailability of risperidone were almost unchanged when oral residence time increased from 0 to 10 min. The calculated sensitivity factor for T_max_, C_max_, AUC_0-∞_, and bioavailability were 0.26, 0.10, 0.11, and 0.11, respectively. All of the values were low. No obvious difference was observed for the C_max_, AUC_0-∞_, and bioavailability of risperidone ODF when oral residence time increased within 10 min based on the calculated value and the graphic trend (**Figure 4**). The results indicated that oromucosal absorption might not be critical to risperidone ODF in short residence time.

### Regional Absorption Prediction

The fraction absorbed of risperidone ODF in the oral cavity at an oral residence time of 2 min, 5 min, and 10 min was predicted to be 7.0%, 11.4%, and 19.5%, respectively. The extent of absorption increased slightly as the oral residence time prolonged. Since risperidone ODF dissolved completely in the oral cavity of Beagle dogs within 2 min, the absorption percentage *via* oral mucosa was almost negligible. Thus, the gastrointestine might be the main absorption route for risperidone ODF. The PBPK modeling of risperidone ODF could be simplified to include ACAT model solely.

The results indicated that the reason for the similar PK profiles following sublingual and supralingual administration of risperidone ODF was that the absorption routes of the two administration sites were the same and they were both mainly absorbed by gastrointestinal tract after entering with saliva although sublingual mucosa has the better permeability and higher vascularization.

### Model Validation

**Figure 5:**
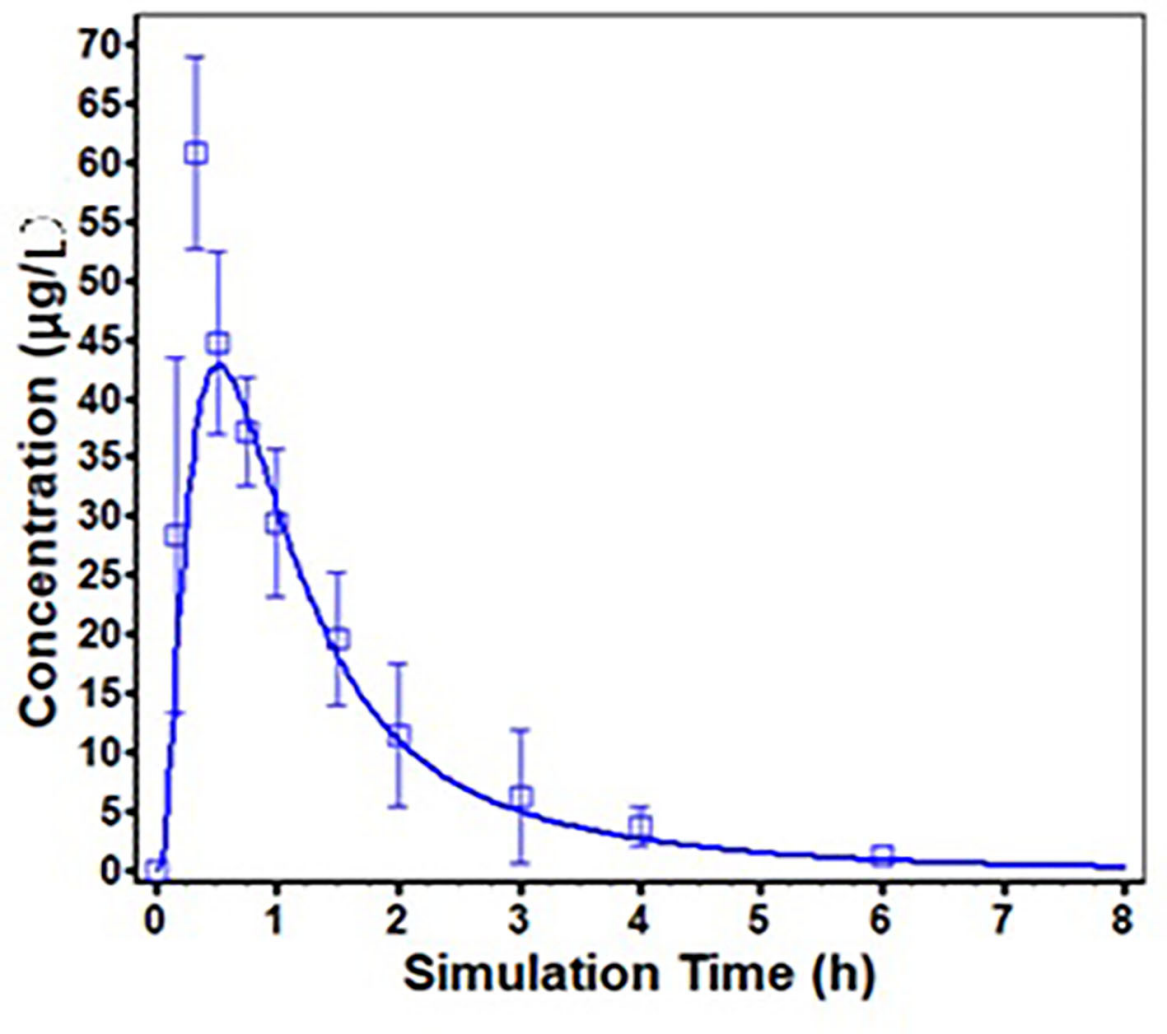
Predicted (solid line) and Observed (square frame, error bars represent one standard deviation from the mean) mean plasma concentration profiles following supralingual administration of 1 mg/body risperidone ODF.

ODFs are usually designed for supralingual administration, and there are no difference of PK profile of risperidone between supralingual and sublingual administration route. Thus, only the PK data of supralingual administration of risperidone ODF was used for the model validation. The *in vivo* absorption of risperidone ODF following supralingual administration was predicted using the ACAT model, and was compared with observed data. The simulated plasma concentration-time profiles are described in **Figure 5**. There is a good match between the predicted plasma concentration-time curve and the observed one.

The predicted and observed main pharmacokinetic parameters are summarized in **Table 3**. The fold errors of T_max_, C_max_, and AUC_0-∞_ for risperidone were 1.17, 1.54, 1.11, respectively. All were within 2-fold, indicating the good prediction of the developed PBPK model for risperidone ODF.

**Table 3:** Comparison of main pharmacokinetic parameters of predicted and observed after supralingual administration of 1 mg/body risperidone ODF.

Parameters	Unit	Predicted	Observed	Fold error
T_max_	h	0.53	0.62	1.17
C_max_	μg/L	38.43	59.38	1.54
AUC_0-∞_	μg/L·h	91.56	101.87	1.11

## Discussion

ODFs can maintain some time of contact in mucosal surface on which they dissolve rapidly and release drug into the saliva. The drugs may be absorbed into the systemic circulation either through oral mucosa, or intestinal mucosa, or both. It was found that the sildenafil ODF was bioequivalent to that of the conventional tablet (Viagra^®^). The plasma concentration-time profiles of sildenafil and the metabolite were nearly superimposable between the two dosage forms (Radicioni et al., 2017). However, after sublingual or buccal administration of the ropinirole ODF, a fast absorption was achieved within 15 min. The bioavailability was dramatically improved by about 7-fold compared to that of oral administration route (Lai et al., 2018). Similar result was also seen in selegiline. The bioavailability of 1.25 mg Zydis selegiline (Zelapar^®^), which was an oral lyophilisate (tablet) that dissolves rapidly when it was put into the oral cavity, was comparable to that of the standard oral tablets of 10 mg. But the principal metabolites were at least 90% lower compared with the oral tablet, leading to a reduction of the dose-related side effect (Poston and Waters, 2007). Therefore, it is important to investigate the mechanism of absorption in the development of these intraoral formulations.

Various models have been developed to investigate the permeability of the compounds in the intraoral formulations across the oral mucosa including *in vitro* models (animal/human tissue model, TR 146 cell culture, EpiOral™, PermeaPad^®^, et al), *in situ* model, *in vivo* pharmacokinetic study, and i*n silico* absorption model (Patel et al., 2012). The *in silico* absorption model integrates the compound and formulation properties as well as physiology and anatomy data, and is thus ideally suitable to investigate the *in vivo* absorption characteristics and mechanism of the intraoral formulations, providing important insights into the factors leading to the different bioavailability.

There are few reports on the application of PBPK modeling to aid the development of the intraoral drug delivery systems. A PBPK model has been built for buprenorphine to predict the pharmacokinetics of buprenorphine sublingual tablets *in vivo* under different dosing strength, guiding the clinical trial design and the rational use of the drug by Kalluri and his co-workers (Kalluri et al., 2017). Due to the lack of oral cavity physiology module in Simcyp™, the inhalation route was used to simulate the portion of sublingual absorbed. The *in vivo* absorption properties and quantitative contribution of each absorption route of buprenorphine were predicted using the validated PBPK model. The prediction of buprenorphine utilized a “top-down” strategy, for which the *in vivo* pharmacokinetic data was used to calculate the contribution percentage of each absorption route based on a great deal of pharmacokinetic data and the general understanding of the absorption mechanism. Additionally, they employed a simple first-order absorption process without the full consideration of the physiological difference between the oral cavity and lung. The oral transmucosal absorption is driven by a complicated partition between saliva and mucosa tissue, permeation through epithelium, absorption into the system circulation, and the portion of the compound swallowed unintentionally. The OCCAT model in GastroPlus™ takes into account the compound dissolution/precipitation in saliva, partition between saliva and mucosa tissue, diffusion through the oral mucosa, and absorption into the blood, as well as the link with the ACAT model to determine gastrointestinal absorption of the swallowed portion. Thus it can directly estimate the oral transmucosal absorption proportion using the physicochemical data of compounds. The predictive capability of the OCCAT model has ever been evaluated by zolpidem sublingual tablets (Intermezzo^®^). The results showed that the OCCAT model well captured the observed pharmacokinetics of zolpidem (R^2^ > 0.9). The estimated zolpidem absorption *via* the oral mucosa was about 18% (Xia et al., 2015). In addition, the validation of the OCCAT model was also conducted for other intraoral drug delivery systems, such as sublingual solution (verapamil), and sublingual tablets (propranolol, asenapine, and nicotine) (Xia et al., 2015). The simulated oral transmucosal absorption portion was comparable with the observed data, except nicotine, a small molecule of low lipophilicity and high solubility, which was underestimated (Xia et al., 2015). Therefore, more data for various kinds of compounds with different physicochemical properties are desired to evaluate and optimize the OCCAT model, so as to improve its utility.

In this study, the combination of OCCAT and ACAT model in the software of GastroPlus was used to investigate the absorption routes of risperidone ODF as well as the potential application of the oral transmucosal delivery. Our results showed the successful application of the OCCAT model to quantify the oromucosal absorption of risperidone, a compound of low solubility and high permeability, providing an example for the development of other ODFs. The OCCAT model has great potential to prospectively predict the intraoral drug products at various stage of the drug development, guide the selection of the lead compounds and dosage forms for oral transmucosal delivery, and replace some preclinical studies as well as clinical trials, which can save much time and cost.

The PBPK model of risperidone was established and validated by pharmacokinetic studies in Beagle dogs. Based on the developed PBPK model, the drug absorption routes were investigated, and the influence of formulation and physiological factors on drug absorption was assessed by parameter sensitivity analysis, which can determine the important physiological factors affecting the bioavailability of dosage forms and help the rational design of formulations. For example, if a drug is mainly absorbed through oral mucosa, we can improve the transport of drugs across the oral mucosa by formulation optimization, such as adding mucoadhesive polymers to prolong the residence time on the mucosal surface, or using permeation enhancer to enhance the drug permeation across the oral mucosa. If a drug is not easily absorbed through the oral mucosa and the gastrointestinal tract is the main absorption site, the formulation only needs to be dispersed in the oral cavity and its dissolution characteristics only needs to meet the requirements of common oral dosage forms. Moreover, in the research and development of the drug, the impact of formulation changes on bioavailability can also be predicted through PBPK, reducing the need for clinical trials and shortening the research process.

This method of PBPK modeling provides some insights for the research of intraoral drug delivery systems such as ODFs, ODTs, sublingual films and mucoadhesive buccal films. For drugs with the possibility of oral mucosa absorption, especially those can be rapidly absorbed through oral mucosa, the absorption model of PBPK which combines the OCCAT model with ACAT model can analyze the impact of dosage forms, formulation and the administration sites of the oral cavity on the oral absorption proportion, and suggest the methods to improve the absorption *via* oral mucosa and bioavailability. The optimal preparation design and clinical drug dosing strategy can be determined through modeling and simulation. Thus, the preclinical studies and clinical trials can be reduced. Finally, simplified drug development and supervision can be expected to achieve.

The results of PBPK modeling showed that the fraction absorbed *via* oral mucosa increased from 7.0% to 19.5% when the residence time changed from 2 min to 10 min while the bioavailability of risperidone was almost unchanged. It is not necessary to increase the residence time in formulation design for the fact that the bioavailability of risperidone cannot be significantly improved by prolonging the residence time in the oral cavity. The rapid dissolution and good compliance are the goals in formulation optimization. The slight change of the residence time caused by the change of the formulation will not affect its bioavailability. As risperidone is mainly absorbed through gastrointestine, the individual differences of oral physiological factors such as the amount of saliva and the flow rate of saliva have little effect on its bioavailability. Therefore, risperidone ODF can be developed according to the requirements of oral dosage forms. The quality indexes such as content, content uniformity, stability and dissolution rate can refer to the requirements of conventional oral dosage forms, and bioequivalence evaluation can choose conventional tablets or ODTs as reference formulations.

The absorption rate and extent of drugs *via* oral mucosa are closely related to the physicochemical properties of the drug including molecular weight, octanol-water partition coefficient, solubility, and ionization constant (Bredenberg et al., 2003; Nicolazzo et al., 2005; Pather et al., 2008). Drugs with fairly good lipophilicity and water solubility are favored to facilitate diffusion across the lipid-rich cytomembrane and the hydrophilic cytoplasm. Furthermore, the drugs in the unionized molecule form can be absorbed across the epithelial cells more effectively (Lam et al., 2014). Therefore, the pKa value, representing the extent of ionization at different pH is of great importance. Risperidone is a small molecule compound which has good lipophilicity and poor water solubility (Khames, 2017). It is a dibasic base with dissociation constants of 8.24 (pKa_1_) and 3.11 (pKa_2_) which is easy to ionize at the oral mucosa (Saibi et al., 2012). For these reasons, it's difficult for risperidone to permeate across the oral mucosa. In addition to the physicochemical properties of the drug, the residence time of the formulation in mucosal surface also has an impact on the oral transmucosal absorption. Risperidone ODF dissolves rapidly in the oral cavity, the residence time of the formulation was within 2 min, which limited the absorption of risperidone *via* oral mucosa.

## Conclusion

The PBPK modeling of risperidone ODF indicated that the majority of the absorption occurred in the gastrointestine, and the percent absorbed *via* oral mucosa was almost negligible as the fact that risperidone ODF dissolved completely in the mouth of Beagle dogs within 2 min. The PBPK mechanistic absorption model which combines OCCAT with ACAT model could be used for the prediction of the *in vivo* absorption to scientifically and rationally guide the development of the ODF product, accelerating the research and development process.

## Data Availability Statement

The datasets generated for this study are available on request to the corresponding authors.

## Ethics Statement

This research was approved by the Animal Management and Ethic Committee of the China State Institute of Pharmaceutical Industry.

## Author Contributions

All authors listed have made substantial, direct, and intellectual contribution to the work and approved it for publication.

## Funding

This work was supported by National Natural Science Foundation of China (81473409), Foundation of Shanghai Science and Technology Commission (18DZ2290500), PDH-SPFDU Joint Research Fund (RHJJ2017-05), and Shanghai Science and Technology Innovation Fund (18140900900).

## Conflict of Interest

The authors declare that the research was conducted in the absence of any commercial or financial relationships that could be construed as a potential conflict of interest.
